# Genome assembly and phylogenomic data analyses using plastid data: Contrasting species tree estimation methods

**DOI:** 10.1016/j.dib.2019.104271

**Published:** 2019-07-27

**Authors:** D.J.P. Gonçalves, B.B. Simpson, G.H. Shimizu, R.K. Jansen, E.M. Ortiz

**Affiliations:** a Department of Integrative Biology, The University of Texas at Austin, 2415 Speedway #C0930, Austin, TX, 78713, USA; b Department of Plant Biology, University of Campinas, 13083-970, Campinas, SP, Brazil; c Genomics and Biotechnology Research Group, Department of Biological Sciences, Faculty of Science, King Abdulaziz University, Jeddah, 21589, Saudi Arabia; d Department of Ecology & Ecosystem Management, Plant Biodiversity Research, Technical University of Munich, Emil-Ramann Strasse 2, Freising, D-85354, Germany

**Keywords:** Data processing, Genome assembly, Phylogenetic analyses, Phylogenetic signal, Tree space

## Abstract

Phylogenomics has become increasingly popular in recent years mostly due to the increased affordability of next generation sequencing techniques. Phylogenomics has sparked interest in multiple fields of research, including systematics, ecology, epidemiology, and even personalized medicine, agriculture and pharmacy. Despite this trend, it is usually difficult to learn and understand how the analyses were done, how the results were obtained, and most importantly, how to replicate the study. Here we present the data and all of the code utilized to perform phylogenomic inferences using plastome data: from raw data to extensive phylogenetic inference and accuracy assessment. The data presented here utilizes plastome sequences available on GenBank (accession numbers of 94 species are available below) and the code is also available at https://github.com/deisejpg/rosids. Gonçalves et al. is the research article associated with the data analyses presented here.

**Table:** Specifications table

Subject area	Biology
More specific subject area	Systematics of angiosperms, plastome evolution
Type of data	Table, PHYLIP, NEXUS, and MARKDOWN formatted files
How data was acquired	Illumina HiSeq. 2500 and Illumina HiSeq 4000
Data format	Raw and Analyzed
Experimental factors	Total genomic DNA isolated and sequenced from fresh or silica dried leaf tissue of sampled species of rosids and genome sequences from NCBI
Experimental features	Illumina reads preprocessing, genome assembly, phylogenetic inference, topology tests, and phylogenetic signal assessment
Data accessibility	Within this article and at https://github.com/deisejpg/rosids
Related research article	Deise J.P. Gonçalves, Beryl B. Simpson, Edgardo M. Ortiz, Gustavo H. Shimizu, Robert K. Jansen Incongruence between gene trees and species trees and phylogenetic signal variation in plastid genes Molecular Phylogenetics and Evolution 10.1016/j.ympev.2019.05.022

**Table:** 

**Value of the data** • The present data provides details about phylogenomic analysis using a set of well-documented pipelines covering analyses from preprocessing Illumina reads to inferring and testing phylogenies using multiple methods of phylogenetic inference • The data introduce the practical use of multispecies coalescent methods using plastid protein-coding genes and could be adjusted and used with molecular data from different molecular markers and organisms • Accessibility to scripts utilized and data files containing the alignments and trees will enhance the replication of the analyses presented

## 1 Data

A dataset comprising 78 plastid protein-coding genes of 94 species of rosids is presented in Table 1. Here we present all the code used in the analysis of this dataset [1], including the scripts used to quality filter, assemble, extract regions of interest, and perform phylogenomic analysis, in a series of tutorial-like files: I. Genome assembly; II. Phylogenetic Analysis; III. Tree space; IV. Phylogenetic Signal. Part of the data was obtained from GenBank (http://www.ncbi.nlm.nih.gov/genbank). Data for and 27 species from groups of rosids that lacked the information on the database were generated using Illumina HiSeq. A total of 657,471,631 million paired-end reads with an average length of 150 bp was generated (Table 2). Despite the interest on extracting and using only the genes from the plastome, the pipeline for genome assembly presented here also separates contigs from the three cellular genomic compartments with a potential for use used in studies that target not only the plastome, but also mitochondria, nuclear ribosomal DNA, and other nuclear markers. The next set of tutorial-like markdown files present the code utilized for preparing alignments and for inferring phylogenies using an array of strategies of data partition and methods of phylogenetic inference. The code used to explore the similarities/dissimilarities between topologies and for the phylogenetic signal calculation is also presented.

**Table 1:** Classification according to APG IV (2016) of samples used in the study (89 samples of rosids, considered here as fabids + malvids, and five of outgroup), voucher information of newly sequenced plastomes and GenBank accession numbers. Bold font indicates plastid genome sequences generated in this study.

Order	Family	Species	Voucher ID	GenBank Accession Numbers
Ingroup				
Brassicales	Brassicaceae	*Brassica napus*		NC_016734
Brassicales	Caricaceae	*Carica papaya*		NC_010323
**Brassicales**	**Moringaceae**	***Moringa oleifera***	**CONN-129179**	MK726020
**Brassicales**	**Salvadoraceae**	***Azima tetracantha***	**CONN00225893**	MK726028
Celastrales	Celastraceae	*Euonymus japonicus*		NC_028067.1
Cucurbitales	Cucurbitaceae	*Citrullus lanatus*		NC_032008
Cucurbitales	Cucurbitaceae	*Cucumis hystrix*		NC_023544
Cucurbitales	Cucurbitaceae	*Gynostemma pentaphyllum*		NC_029484
Fabales	Fabaceae	*Cicer arietinum*		NC_011163
Fabales	Fabaceae	*Inga leiocalycina*		NC_028732
Fabales	Fabaceae	*Lupinus luteus*		NC_023090
**Fabales**	**Polygalaceae**	***Polygala alba***	**TEX-DJPG731**	MK726019
Fagales	Betulaceae	*Ostrya rehderiana*		NC_028349
Fagales	Fagaceae	*Castanea mollissima*		NC_014674
Fagales	Juglandaceae	*Juglans regia*		NC_028617
Geraniales	Francoaceae	*Francoa sonchifolia*		NC_021101
Geraniales	Francoaceae	*Melianthus villosus*		NC_023256
Geraniales	Francoaceae	*Viviania marifolia*		NC_023259
Geraniales	Geraniaceae	*Erodium rupestre*		NC_030719
Geraniales	Geraniaceae	*Geranium palmatum*		NC_014573
Geraniales	Geraniaceae	*Hypseocharis bilobata*		NC_023260
Geraniales	Geraniaceae	*Monsonia speciosa*		NC_014582
Geraniales	Geraniaceae	*Pelargonium alternans*		NC_023261
Malpighiales	Chrysobalanaceae	*Chrysobalanus icaco*		NC_024061
Malpighiales	Chrysobalanaceae	*Hirtella racemosa*		NC_024060
Malpighiales	Erythroxylaceae	*Erythroxylum novogranatense*		NC_030601
Malpighiales	Euphorbiaceae	*Ricinus communis*		NC_016736
**Malpighiales**	**Malpighiaceae**	***Galphimia angustifolia***	**TEX-DJPG803**	MK726010
Malpighiales	Salicaceae	*Salix babylonica*		NC_028350
Malvales	Malvaceae	*Gossypium turneri*		NC_026835
Malvales	Malvaceae	*Hibiscus syriacus*		NC_026909
Malvales	Malvaceae	*Theobroma cacao*		NC_014676
Malvales	Thymelaeaceae	*Aquilaria sinensis*		NC_029243
**Myrtales**	**Alzateaceae**	***Alzatea verticillata***	**K-TNV548**	MK726006
**Myrtales**	**Combretaceae**	***Laguncularia racemosa***	**CONN00225898**	MK726017
**Myrtales**	**Combretaceae**	***Terminalia guyanensis***	**UEC-GHS1070**	MK726027
**Myrtales**	**Lythraceae**	***Heimia apetala***	**CONN00225896**	MK726012
Myrtales	Lythraceae	*Lagerstroemia guilinensis*		NC_029885
Myrtales	Lythraceae	*Lagerstroemia fauriei*		NC_029808
Myrtales	Lythraceae	*Lagerstroemia indica*		NC_030484
Myrtales	Melastomataceae	*Blakea schlimii*		NC_031877
Myrtales	Melastomataceae	*Henriettea barkeri*		NC_031880
**Myrtales**	**Melastomataceae**	***Memecylon pauciflorum***	**K-TNV679**	MK726029
Myrtales	Melastomataceae	*Miconia dodecandra*		NC_031882
Myrtales	Melastomataceae	*Rhexia virginica*		NC_031886
**Myrtales**	**Melastomataceae**	***Tibouchina urvilleana***	**CONN00225897**	MK726030
Myrtales	Myrtaceae	*Allosyncarpia ternata*		NC_022413
Myrtales	Myrtaceae	*Corymbia eximia*		NC_022409
Myrtales	Myrtaceae	*Eucalyptus globulus*		NC_008115
Myrtales	Myrtaceae	*Eugenia uniflora*		NC_027744
**Myrtales**	**Myrtaceae**	***Heteropyxis natalensis***	**K-MFF s.n.**	MK726014
Myrtales	Myrtaceae	*Psidium guajava*		NC_033355
Myrtales	Myrtaceae	*Stockwellia quadrifida*		NC_022414
**Myrtales**	**Myrtaceae**	***Xanthostemon chrysanthus***	**K-TNV684**	MK726024
Myrtales	Onagraceae	*Ludwigia octovalvis*		NC_031385
Myrtales	Onagraceae	*Oenothera argillicola*		NC_010358
Myrtales	Onagraceae	*Oenothera grandiflora*		NC_029211
Myrtales	Onagraceae	*Oenothera oakesiana*		NC_029212
Myrtales	Onagraceae	*Oenothera villaricae*		NC_030532
**Myrtales**	**Penaeaceae**	***Saltera sarcocolla***	**CONN00225892**	MK726025
**Myrtales**	**Vochysiaceae**	***Callisthene erythroclada***	**UEC-DG439**	MK726008
**Myrtales**	**Vochysiaceae**	***Erisma bracteosum***	**UEC-GHS937**	MK726009
**Myrtales**	**Vochysiaceae**	***Erismadelphus exsul***	**BRLU-SB751**	MK726007
**Myrtales**	**Vochysiaceae**	***Korupodendron songweanum***	**BRLU-SB3491**	MK726013
**Myrtales**	**Vochysiaceae**	***Qualea grandiflora***	**UEC-DG382**	MK726022
**Myrtales**	**Vochysiaceae**	***Ruizterania albiflora***	**UEC-TM258**	MK726023
**Myrtales**	**Vochysiaceae**	***Salvertia convallariodora***	**TEX-DJPG569**	MK726026
**Myrtales**	**Vochysiaceae**	***Vochysia acuminata***	**TEX-DJPG150**	MK726031
Oxalidales	Oxalidaceae	*Averrhoa carambola*		NC_033350
**Oxalidales**	**Oxalidaceae**	***Oxalis drummondii***	**TEX-DJPG722**	MK726021
Rosales	Cannabaceae	*Cannabis sativa*		NC_026562
Rosales	Elaeagnaceae	*Elaeagnus macrophylla*		NC_028066
Rosales	Moraceae	*Ficus racemosa*		NC_028185
Rosales	Moraceae	*Morus indica*		NC_008359
Sapindales	Sapindaceae	*Acer morrisonense*		NC_029371
Sapindales	Anacardiaceae	*Anacardium occidentale*		NC_035235.1
Sapindales	Anacardiaceae	*Mangifera indica*		NC_035239.1
Sapindales	Burseraceae	*Boswellia sacra*		NC_029420
Sapindales	Meliaceae	*Azadirachta indica*		NC_023792
Sapindales	Rutaceae	*Citrus aurantiifolia*		NC_024929
Sapindales	Rutaceae	*Zanthoxylum piperitum*		NC_027939
**Sapindales**	**Sapindaceae**	***Dimocarpus longan***	**UCONN-201400014**	MK726005
Sapindales	Sapindaceae	*Dipteronia sinensis*		NC_029338
Sapindales	Sapindaceae	*Litchi chinensis*		NC_035238.1
Sapindales	Sapindaceae	*Sapindus mukorossi*		NC_025554
Vitales	Vitaceae	*Tetrastigma hemsleyanum*		NC_029339
Vitales	Vitaceae	*Vitis vinifera*		NC_007957
**Zygophyllales**	**Krameriaceae**	***Krameria bicolor***	**TEX-JN14-09-03-1**	MK726015
**Zygophyllales**	**Krameriaceae**	***Krameria lanceolata***	**TEX-JN6-IV-2015-1**	MK726016
**Zygophyllales**	**Zygophyllaceae**	***Guaiacum angustifolium***	**TEX-BBS20-IV-2015-1**	MK726011
**Zygophyllales**	**Zygophyllaceae**	***Larrea tridentata***	**TEX-JN14-08-24-1**	MK726018
Outgroup				
Caryophyllales	Caryophyllaceae	*Silene capitata*		NC_035226
Apiales	Araliaceae	*Aralia undulata*		NC_022810
Solanales	Solanaceae	*Nicotiana tabacum*		NC_001879.2

**Table 2:** Summary of output for Illumina sequencing of the 23 complete and the 4 draft plastomes.

Species	Total # Reads	Plastid reads	Average fold coverage^a^
*Laguncularia racemosa*	26,332,422	992,869	758.64
*Terminalia guyanensis*	28,526,166	335,902	411.37
*Heimia apetala*	27,057,568	3480,902	3630.02
*Memecylon pauciflorum*	29,404,628	1,776,470	2078.67
*Tibouchina urvilleana*	51,945,408	1,565,235	1321.25
*Heteropyxis natalensis*	23,511,880	437,572	620.57
*Saltera sarcocolla*	42,509,058	1,975,497	1589.48
*Callisthene erythroclada*	13,523,069	1,475,969	1679.93
*Erisma bracteosum*	28,714,784	740,005	872.25
*Qualea grandiflora*	14,026,674	315,666	361.56
*Korupodendron songweanum*	26,380,860	621,934	697.51
*Ruizterania albiflora*	13,416,345	346,923	395.21
*Salvertia convallariodora*	13,719,886	1,131,571	1262.57
*Vochysia acuminata*	14,190,315	1,404,610	1588.55
*Azima tetracantha*	19,768,664	588,345	676.25
*Moringa oleifera*	35,924,836	3,914,845	3054.87
*Dimorcarpus longan*	39,914,336	1,125,881	870.47
*Galphimia angustifolia*	20,773,804	1,235,549	1449.65
*Oxalis drummondii*	30,898,958	1,231,010	1698.62
*Krameria bicolor*	20,882,876	1,032,768	1236.57
*Krameria lanceolata*	20,840,984	191,073	206.57
*Guaiacum angustifolium*	27,224,600	131,532	143.81
*Larrea tridentata*	23,104,094	2,094,664	2797.51
*Alzatea verticillata* (reads mapped to S. sarcocolla)	11,665,088	51,656	61.91
*Xanthostemon chrysanthus* (reads mapped to *H. natalensis*)	9,809,306	51,304	58.28
*Polygala alba* (reads mapped to *G. angustifolia*)	30,134,340	125,019	101.66
*Erismadelphus exsul* (reads mapped to *K. songweanum*)	13,270,682	286,659	368.03

a Coverage calculation was performed in bbmap (BBTools package, available at https://jgi.doe.gov/data-and-tools/bbtools/) with the option “covstats”. Reference index was built with the 23 complete plastomes with each representing a scaffold. The average fold coverage was calculated by mapping reads to the reference index and values were taken from the scaffold correspondent to each species. For species with incomplete plastomes (marked in bold) we used the closely related species as the scaffold indicated within the parentheses.

## 2 Experimental design, materials and methods

### 2.1 Data preprocessing and genome assembly

For the 27 samples for which data were generated, leaf tissue was ground and total genomic DNA was isolated using DNeasy Plant Mini Kit (Qiagen) according to the manufacturer's protocol or a modified version of [2] described in Ref. [3]. The DNA was quantified using a Qubit Fluorometric Quantitation (Thermo Fisher) instrument and was sequenced at the Genome Sequencing and Analysis Facility (GSAF) at The University of Texas at Austin. Two species were kindly provided by The Royal Botanic Gardens, Kew, DNA bank (https://www.kew.org/data/dnaBank/).

Once the reads were available, the genome assembly pipeline was used to remove adaptors and PHIX, for quality trimming, and for genome assembly.

### 2.2 Phylogenetic inference

After gathering sequences of plastid protein-coding genes, the alignments and phylogenetic inference were performed. The code used to prepare the alignments using MAFFT [4] and MACSE [5] as well as the scripts used to infer phylogenies using Maximum Likelihood (ML), IQ-TREE [6], and Multispecies Coalescent (MSC) methods, SVDquartets [7], and ASTRAL-II [8] is presented in phylogenetic analysis pipeline.

### 2.3 Calculating distances of tree topologies and phylogenetic signal

Commented scripts present how the inferred phylogenies were further explored. First, Robinson-Foulds and Kendall-Colijn algorithms implemented in the R package TREESPACE [9] were used to visualize the distances of species trees and between species trees and gene trees inferred. The code used is available at tree space. Lastly, five taxa from different taxonomic levels that had alternative placements were selected for a set of measurements of gene-wise and site-wise log-likelihood support of alternative topologies phylogenetic signal.
